# Influence of Prenatal Exposure to Mercury, Perceived Stress, and Depression on Birth Outcomes in Suriname: Results from the MeKiTamara Study

**DOI:** 10.3390/ijerph17124444

**Published:** 2020-06-20

**Authors:** Anisma R. Gokoel, Wilco C. W. R. Zijlmans, Hannah H. Covert, Firoz Abdoel Wahid, Arti Shankar, M. Sigrid MacDonald-Ottevanger, Ashna D. Hindori-Mohangoo, Jeffrey K. Wickliffe, Maureen Y. Lichtveld, Emily W. Harville

**Affiliations:** 1 Scientific Research Center Suriname, Academic Hospital Paramaribo, Paramaribo, Suriname; wilco.zijlmans@uvs.edu (W.C.W.R.Z.); fabdoelw@tulane.edu (F.A.W.); m.sigridmacdonald@gmail.com (M.S.M.-O.); 2 Faculty of Medical Sciences, Anton de Kom University of Suriname, Paramaribo, Suriname; 3 Department of Environmental Health Sciences, School of Public Health and Tropical Medicine, Tulane University, New Orleans, LA 70112, USA; ashna.mohangoo@perisur.org (A.D.H.-M.); jwicklif@tulane.edu (J.K.W.); mlichtve@tulane.edu (M.Y.L.); 4 Department of Biostatistics and Data Science, School of Public Health and Tropical Medicine, Tulane University, New Orleans, LA 70112, USA; sarti@tulane.edu; 5 Department of Medical Microbiology, Academic University Medical Center, 1105 AZ Amsterdam, The Netherlands; 6 Foundation for Perinatal Interventions and Research in Suriname (Perisur), Paramaribo, Suriname; 7 Department of Epidemiology, School of Public Health and Tropical Medicine, Tulane University, New Orleans, LA 70112, USA; eharvill@tulane.edu

**Keywords:** mercury exposure, maternal depression, perceived stress, Apgar score, birthweight, preterm birth, adverse birth outcomes, Suriname

## Abstract

Prenatal exposure to mercury, stress, and depression may have adverse effects on birth outcomes. Little is known on the influence of chemical and non-chemical stressors on birth outcomes in the country of Suriname. We assessed the influence of prenatal exposure to mercury, perceived stress, and depression on adverse birth outcomes in 1143 pregnant Surinamese women who participated in the Caribbean Consortium for Research in Environmental and Occupational Health-MeKiTamara prospective cohort study. Associations between mercury (≥1.1 μg/g hair, USEPA action level/top versus bottom quartile), probable depression (Edinburgh Depression Scale ≥12), high perceived stress (Cohen’s Perceived Stress Scale ≥20), and adverse birth outcomes (low birthweight (<2500 g), preterm birth (<37 completed weeks of gestation), and low Apgar score (<7 at 5 min)) were assessed using bivariate and multivariate logistic regressions. Prevalence of elevated mercury levels, high perceived stress, and probable depression were 37.5%, 27.2%, and 22.4%, respectively. Mercury exposure was significantly associated with preterm birth in the overall study cohort (OR 2.47; 95% CI 1.05–5.83) and perceived stress with a low Apgar score (OR 9.73; 95% CI 2.03–46.70). Depression was not associated with any birth outcomes. These findings can inform policy- and practice-oriented solutions to improve maternal and child health in Suriname.

## 1. Introduction

Maternal exposure to chemical and non-chemical stressors may result in adverse health effects for mother and child. Exposures to mercury, stress, or depression that may result in adverse birth outcomes and long-term neurodevelopmental effects in children are of particular concern. Women prenatally exposed to mercury are more likely to deliver infants who have a low birthweight or who are small-for-gestational age [1], as well as infants with a smaller head circumference, lower Apgar score at 5 min, and shorter umbilical cord length [2]. In addition, prenatal exposure to mercury has also been associated with irreversible neurodevelopmental deficits [3]. High perceived stress during pregnancy and prenatal depression has been associated with low birthweight (LBW) [4,5], preterm birth (PTB) [4,5,6], and a low Apgar score [7]. Noteworthy is that women with high perceived stress levels have a greater risk of developing prenatal and postnatal depression [8,9].

There is accumulating evidence that combined exposure to chemical and non-chemical stressors has a synergistic effect on maternal and child health [10,11,12]. A significant negative effect on the maternal cortisol slope was found in pregnant women exposed to high stress and mercury levels above the median as compared with women experiencing high stress levels but mercury levels below the median [10]. In addition, a recent review portrayed the synergistic effect of air pollution and stress during pregnancy on child outcomes, especially asthma and wheezing [12].

Consequences of adverse birth outcomes include mortality, delay in neurodevelopment for infants and children, and emotional and economic costs for families [5]. Adverse birth outcomes are proportionally higher in low- and middle-income countries (LMICs) where the incidence of LBW can be as high as 30%, as compared with 6% in developed countries [13]. Further, preterm-born children are more prevalent in LMICs (12%) than in developed countries (9%) [14].

Suriname is a middle-income country situated on the northeastern coast of South America. The majority of its 541,638 inhabitants (66%) reside in urban areas, and the remainder in rural areas (21%) and the Amazonian interior rainforest (13%) [15]. Suriname has a multi-ethnic population consisting of people of Asian (41.1%; Hindustani and Javanese), African (37.4%; Tribal People and Creoles) and “Other” descent (21.0%; Mixed, Amerindians, Chinese, and Caucasians) [15]. Preliminary results of the Perinatal and Infant Mortality Survey in Suriname (POPZiS) of all infants born between September 2010 and December 2012 (*n* = 5371 live births) indicated a neonatal mortality rate of 12.9%. Most of these neonates had a birthweight of less than 2500 g (71.2%) and were born prematurely (67.7%) [16].

In Suriname, mercury is mainly used in artisanal and small-scale goldmining in the Amazonian interior. Elemental mercury used in goldmining pollutes freshwater bodies where it ultimately accumulates in fish, the main protein source for the interior population. A study in Suriname found mercury levels above the European Union standard for human consumption of 0.5 μg g^−1^ and the US EPA standard for human consumption of 1 μg g^−1^ in collected piscivorous fish (red-eyed piranha/Serrasalmus rhombeus and aimara/Hoplias aimara) [17]. High levels of mercury were also found in hair samples of women living in the interior [18]. In addition, a pilot study showed high levels in the hair of pregnant women and their newborns from Paramaribo, the capital city [19]. As there is no goldmining in Paramaribo, this finding may be related to occupational exposure to mercury.

Findings of the Caribbean Consortium for Research in Environmental and Occupational Health (CCREOH)-MeKiTamara study, an ongoing prospective cohort study focused on maternal and child health in Suriname, showed that prenatal depression and perceived stress are important health issues among pregnant women in Suriname. The prevalence of high perceived stress in participants during the first two trimesters of pregnancy was 27.2%, while probable depression was 22.4% during the first or second trimester. Women experiencing high stress levels during the first two trimesters had almost twice the odds of having probable depression during the third trimester. Lower educational level and age (≥20 years) were significantly associated with probable depression during the third trimester [20].

Despite the potentially high levels of exposure to mercury, perceived stress, and depression and the high incidence of low birthweight and preterm birth in Suriname, no studies have examined the influence of chemical and non-chemical stressors on birth outcomes. As pregnant women are typically exposed to both kinds of stressors, it is important to not only examine the separate effects of the exposures, but also the combined effect on birth outcomes. The aim of this study is to examine the influence of perceived stress, depression, and mercury on birth outcomes in Suriname. Towards this end, the relation between hair mercury levels, the Cohen Perceived Stress Scale, and the Edinburgh Depression Scale, and the birth outcomes of low birthweight, low Apgar score, and preterm birth are assessed in the MeKiTamara cohort.

## 2. Materials and Methods

### 2.1. Study Design

The MeKiTamara study is a prospective environmental epidemiologic cohort study. MekiTamara means “creating a mother and child’s tomorrow” in Sranang tongo, Suriname’s lingua franca. Pregnant women were recruited in three regions of Suriname: the capital city of Paramaribo, the agricultural district of Nickerie, and the Amazonian interior.

### 2.2. Study Population

From December 2016 to July 2019, eligible pregnant women were recruited from (1) Paramaribo, at four hospitals (the Academic Hospital Paramaribo, Diakonessen Hospital, ‘s-Lands Hospital, Saint Vincentius Hospital) and at prenatal clinics and midwife facilities of the regional health department; (2) Nickerie, at the Mungra Medical Centre Hospital and at regional health department clinics and facilities; and (3) the Amazonian interior, at multiple health care clinics of the Medical Mission Primary Health Care Suriname. Women were eligible if they were 16 years or older, spoke Dutch, Saramaccan, or Trio, had a singleton gestation, were planning to give birth at one of the study sites, and provided written informed consent/assent. A total of 1143 women were included in this study.

### 2.3. Data Collection

Interviewer-assisted surveys (including standardized questionnaires and a series of demographic questions) were conducted by trained recruiters using encrypted iPads. Survey data were uploaded to Research Electronic Data Capture (REDCap). REDCap is a secure web application for building and monitoring online surveys and databases, and can be used online or offline to collect data for research [21]. The standardized questionnaires were collected at two time points during pregnancy (1st or 2nd trimester and 3rd trimester). During the 3rd trimester, data were collected from 971 of the 1143 participants that originally enrolled. This difference of 172 participants is accounted for as follows: 33 participants (2.9%) had a miscarriage/intrauterine fetal death, 3 participants (0.3%) had multiple gestations, 88 participants (7.7%) were lost to follow-up (moved to another county or unreachable), and 48 participants (4.2%) withdrew.

Questionnaires included Cohen’s Perceived Stress Scale and the Edinburgh Depression Scale. Hair was sampled late in the first trimester or early in the second trimester by cutting 1.5 g of hair as close as possible to the scalp using sterilized scissors. Samples were immediately stored in a sealed plastic pouch and refrigerated. They were then sent for storage at 4 °C until analysis at the National Zoological Collection of Suriname/Center for Environmental Research Lab at the Anton de Kom University of Suriname. Selected hair samples were evaluated at two laboratories and went through extensive quality assurance/quality control to ensure that the mercury being measured had been deposited within the hair shaft matrix and was not an external contaminant [22].

### 2.4. Exposures and Covariates

#### 2.4.1. Demographic Data

Demographic variables were categorized into the following groups: age (16–19, 20–34, or ≥35 years), marital status (married/cohabitating, living alone/single), household income (<3000, or ≥3000 SRD (USD 400)), educational level (no, primary or lower secondary, upper secondary or tertiary), race/ethnicity with African descent (Creole, Tribal), Asian descent (Hindustani, Javanese), or other (Caucasian, Indigenous, Mixed)), parity (no previous live birth, 1 previous live birth, more than 1 previous live birth), and region: urban (Paramaribo, Wanica), rural (Commewijne, Saramacca, Para, Nickerie and Coronie), and the interior (Marowijne, Brokopondo and Sipaliwini).

#### 2.4.2. Hair Mercury

The total hair mercury concentration was reported in units of μg/g. The cut-off for elevated mercury levels was set at ≥1.1 μg/g according to the US Environmental Protection Agency (US EPA) action level. In addition, mercury levels were divided into quartiles. Hair mercury levels and blood mercury levels for this population were highly correlated (Spearman’s r = 0.83, 95% CI 0.73–0.89, *p* < 0.001) [22]. Hair mercury concentrations were used as an indicator of exposure in this study because most of the mercury in hair is methylmercury, the main form of mercury found in fish from gold-mining [23]. In addition, hair mercury is a better measure of long-term exposure than blood [24].

#### 2.4.3. Perceived Stress

The Cohen Perceived Stress Scale (PSS-10) includes 10 items about the degree of experiencing stress due to having no control over things, nervousness, and not feeling confident about their ability to cope with things during the past month. There are five response options: 0 for never, 1 for almost never, 2 for sometimes, 3 for fairly often, and 4 for very often. The total score ranges from 0 to 40 points, 0 indicates the lowest stress level and 40 the highest stress level. In this study, the cut-off point was set at the 75th percentile (20) for a positively skewed distribution. Since the majority of the scores in the study population were low, we compared the top 25% with the rest of the data. In our earlier study of the MeKiTamara cohort, 27.2% of the 1143 pregnant women scored between 20 and 40 on the Cohen’s Perceived Stress Scale during the first or second trimester, indicating high perceived stress levels [20].

#### 2.4.4. Depression

The Edinburgh Postnatal Depression Scale assesses postnatal depression, but has been validated for use prenatally. During the prenatal phase, it is known as the Edinburgh Depression Scale (EDS) [25]. The EDS includes 10 statements concerning depressive and anxiety-related symptoms on a four-point Likert scale: 0 = yes, very often; 1 = yes, mostly; 2 = no, not often; and 3 = no, not at all. The total depression sum score of all statements ranges from 0 to 30 points. A higher total depression score indicated a higher risk of probable depression. In this study, a cut-off point of ≥12 points was used to indicate probable depression as defined by the scale, compared with a score of 0–11 points for no depression [26]. Previous findings of the MeKiTamara cohort showed that 22.4% of participants scored ≥12 during the first or second trimester on the Edinburgh Depression Scale, indicating probable depression [20].

#### 2.4.5. Birth Outcomes

Information was obtained from labor and delivery books completed by midwives in the hospitals, prenatal clinics, or midwife facilities where the baby was born. Low birthweight was defined as birthweight <2500 g, preterm birth as birth before 37 completed weeks of gestation, and low Apgar score as <7 at 5 min.

### 2.5. Data Analysis

Total mercury analysis was conducted with cold vapor atomic absorption spectrometry (CVAAS) (Bacharach, Inc., New Kensington, PA, USA). Questionnaire and demographic data were analyzed using SPSS version 20 (IBM Corp., Armonk, NY, USA). Bivariate logistic regression was used to determine the association between demographic factors and the exposure variables and between demographic variables and the birth outcomes. Bivariate logistic regression analysis was also conducted to determine the association between the exposure variables and LBW, PTB, and low Apgar score. We examined the exposure variables as both continuous and categorical variables. Since the results were similar for both types of variables, we decided to use categorical variables for ease of presentation and interpretability. Multivariable logistic regressions, adjusted for covariates that were significantly associated with both the exposure variables and with the birth outcome in question, were conducted. Interaction models were performed to examine the combined association between high mercury levels and high stress/depression levels on birth outcomes. A *p*-value ≤ 0.05 was considered significant.

### 2.6. Ethical Considerations

This study was approved by the Institutional Review Board (IRB) of Tulane University and the Medical Ethical Commission of Suriname’s Ministry of Health (VG 023-14). All included women (18+) provided written informed consent, and assent was obtained from women who were 16 or 17 years old.

## 3. Results

### 3.1. Population Demographics

Demographic characteristics are presented in Table 1. The age of the pregnant women varied between 16–49 years with a mean age of 28 years (SD 6.43). The majority were in the age category 20–34 years (71.7%) and the largest group was of African descent (Creole or Tribal) (45.5%), followed by women of Asian descent (29.2%) (Hindustani and Javanese). Most of the women had a household income <SRD 3000 (equivalent to USD 400) (66.8%), and had no, primary, or lower secondary/vocational education (57.6%). A total of 87.5% of the women were married/cohabitating, had ≥2 previous live births (38.9%), and were living in the urban areas of the country (57.4%).

### 3.2. Mercury

Table 2 shows the perceived stress, depression, and demographic characteristics of the study population according to mercury levels. Overall, 37.5% of participants had a hair mercury level ≥1.1 μg/g, indicating elevated levels. The mean, median, and range of hair mercury were, respectively: 1.857, 0.826, and 0.00–31.91 μg/g. Participants aged 16–19 years (OR 1.93; 95% CI 1.27–2.93) or 35+, (OR 1.60; 95% CI 1.08–2.36) who were of African descent (OR 1.43; 95% CI 1.01–2.02) or Caucasian, Indigenous or Mixed (“other”) (OR 2.51; 95% CI 1.73–3.64) were more likely to have elevated mercury levels compared with participants aged 20–34 years and those of Asian descent. Women with elevated mercury levels had lower household incomes (OR 2.67; 95% CI 1.88–3.78), a lower educational level (OR 4.02; 95% CI 2.93–5.52), were more likely to be married/cohabitating (OR 2.29; 95% CI 1.35–3.89), and to have ≥2 previous live births (OR 1.53 95% CI 1.10–2.13) compared with women with higher household incomes, higher educated, unmarried/single, and with no previous live births. Women from the interior were at a significant higher risk (OR 47.76; 95% CI 24.86–91.74) of having elevated mercury levels compared with women from rural areas. Perceived stress (*p* = 0.097) and depression (*p* = 0.355) were not significantly associated with mercury levels. In a previous study, stress and depression were positively associated in this cohort [20].

### 3.3. Association between Mercury, Perceived Stress, Depression and Birth Outcomes

The average birthweight of the infants was 3014.51 g (SD 584.70), the average gestational age at delivery was 38 weeks (SD 2.44), and the average Apgar score was 9.45 (SD 1.64). Perceived stress was not significantly associated with low birthweight (OR 1.19; 95% CI 0.79–1.80) or preterm birth (OR 1.27; 95% CI 0.86–1.87), but it was associated with a low Apgar score; women with high perceived stress levels had a twofold higher risk of having a baby with a low Apgar score (OR 2.05; 95% CI 0.99–4.25; *p* = 0.05). Neither depression nor high mercury levels showed an association with low birthweight, preterm birth, or low Apgar score (Table 3).

Women aged 35+ (OR 3.15; 95% CI 1.37–7.24), lower educated (OR 2.62; 95% CI 1.11–6.18), and who had no previous live births (OR 3.16; 95% CI 1.29–7.73) had a higher risk of having an infant with a low birthweight than women aged 20–34 years, higher educated, and with a history of ≥2 births (Table 4). In the multivariable analysis, mercury was significantly associated (*p* = 0.039) with PTB in the overall study population (OR 2.47; 95% CI 1.05–5.83) (Table 5) and perceived stress (*p* = 0.004) with a low Apgar score (OR 9.73; 95% CI 2.03–46.70) (Table 6). There were no consistent significant interactions between mercury and the stress/depression variables with birth outcomes.

## 4. Discussion

Over one out of three pregnant Surinamese women enrolled in the MeKiTamara study had elevated hair mercury levels, one out of four had high perceived stress levels, and one out of five had probable depression as defined by the EDS scale during early pregnancy. Hair mercury levels (first versus fourth quartile) were significantly associated with preterm birth. Older age (35+), primiparity, and lower educational level were significantly associated with low birthweight. Women with high perceived stress had almost tenfold higher odds of an infant with a low Apgar score.

We found a statistically significant association between mercury exposure and preterm birth in the overall study cohort. This is in line with another study that showed a significant relation of high hair mercury levels of pregnant women with preterm birth [27]. We did not find an association between mercury exposure and low Apgar score or birthweight, a finding similar to other studies examining chemical stressors and birth outcomes [28,29,30,31].

In the MeKiTamara participants, we see an extremely high likelihood (OR 47.76) of elevated hair mercury levels in women from the interior compared with women from rural areas. Our results also showed an increased risk (OR 2.51) for high mercury levels in the “other” ethnicity group. After splitting the “other” group into the sub-groups, we noticed that 54% of the participants were Indigenous women, of which, 62% had mercury levels in the fourth quartile. Further, 94% of the Indigenous women with elevated mercury levels were living in the interior of Suriname. After limiting our analysis to the interior participant subcohort only, we did not see any significant association between mercury levels and adverse birth outcomes. A reason for this may be the fact that the majority of the participants had elevated mercury levels, and the group with low exposure was too small to see an effect. It is known that mercury is used for artisanal goldmining in the interior, and that the Indigenous Peoples mainly subsist on fish consumption [18]. Fish consumption is thought to contribute to high levels of mercury, which in turn may contribute to adverse birth outcomes [28] and poor mental health [32].

We found perceived stress to be significantly associated with a low 5-min Apgar score. A study which included women from urban and rural health centers in Iran, and using the PSS-14 scale to assess perceived stress, showed similar results. Women who perceived high levels of stress, and concern about feelings and interpersonal relationships had significantly higher scores for perceived stress which was associated with lower Apgar scores [33]. As our MeKiTamara women had almost tenfold higher risks on perceiving high stress levels during the third trimester of pregnancy, this may explain the higher risk on the low 5-min Apgar score [20].

Furthermore, our results showed that older age (35+), primiparity, and lower educational level were significantly positively associated with low birthweight. A systematic review of 41 studies found similar results regarding the association between primiparity and low birthweight [34]. Infants of nulliparous mothers had an unadjusted reduction estimate of 280 g in birthweight compared with multipara mothers. Similar to our study, studies conducted in Lithuania [35] and Indonesia [36] reported older women (>35 years) to be at greater risk for low birthweight. Other underlying causes may explain the fact that older MeKiTamara women had a greater risk of low birthweight, such as chronic diseases or general health status.

We saw a greater risk of low birthweight for women with lower education. As education and income are associated with each other and almost 70% of our participants had lower household incomes, this may be an explanation of the higher risks of low birthweight in lower-educated women. Further, their nutritional status may contribute to low birthweight, assuming that women with a low education and income are less knowledgeable about and maybe cannot afford to buy healthy food. This is confirmed by a study in South Carolina which showed an association between stress and low birthweight, modified by neighborhood, with infants of high poverty neighborhoods being more at risk for low birthweight [37].

We did not find an association between depression and low birthweight, preterm birth, and low Apgar score. A review study looking at the association between prenatal depression and low birthweight and preterm birth between 1977 and 2013 reported less than a quarter of the 50 studies looking at preterm birth and depression to be significantly associated, and more than half of the 33 studies assessing the association between (low) birthweight to be significantly associated with prenatal depression [5]. Non-significant results were more likely in spontaneous preterm birth. This may explain our findings, as our data were limited to whether the infant was born prematurely or not.

To our knowledge, this is the first study in Suriname that assesses the influence of non-chemical chemical stressors on birth outcomes, and the first to assess the combined effect of both chemical and non-chemical stressors on birth outcomes. Our adequate sample size, the even representation of the different ethnicities in Suriname, the three recruitment regions, and the range of socio-demographic factors enhance the generalizability to the Surinamese population. Although the questionnaires used in this study were not specifically validated for Suriname, they are standardized and have been used in other LMICs. Further, as perceived stress and probable depression were measured with a scale and not assessed by a mental health specialist, our results do not equate to a clinical diagnosis. However, because the EDS does not contain somatic symptoms and is validated for use during pregnancy, it is a robust scale and does not produce artificially high scores [5]. Finally, our cross-sectional mercury analysis may be a possible limitation; multiple measurements during gestation might better assess the association with birth outcomes.

## 5. Conclusions

Our results suggest that while mercury was not significantly associated with birthweight and low Apgar score, it may affect preterm birth. This gives more support to the idea that the Surinamese government should strengthen and enforce laws regarding mercury use, and ensure adequate monitoring of mercury use in goldmining to protect interior inhabitants, especially pregnant women and their infants. The population should also be well-informed of the adverse health effects of mercury exposure during pregnancy on mother and child. A recommendation would be to provide information about mercury levels in fish and the recommended levels of fish consumption during pregnancy. Since we did not examine fish intake, nor the levels of omega 3-fatty acids for the consumed fish in this study, we recommend further research. Regarding the high levels of perceived stress and depression as measured by questionnaires, our results call for effective and timely clinical prenatal screening by general practitioners, gynecologists, or midwives at prenatal visits. As this is the first study examining the influence of chemical and non-chemical stressors in Suriname, this research makes a significant contribution to the knowledge about maternal and child health in the country. Further research is needed to examine the association between mercury, depression, and birth outcomes. In addition, other risk factors for adverse birth outcomes such as substance use, nutritional status, chronic diseases, and prenatal care utilization should be considered. It is important to keep in mind that this study did not assess the association of prenatal mercury exposure with neurodevelopment outcomes in children in the cohort: this research is ongoing.

## Figures and Tables

**Table 1:** Demographic characteristics of the study population.

Characteristic	Total *n* (%)
Total	1143 (100)
Age (years)	
Mean (SD)	28 (6.43)
16–19	144 (12.6)
20–34	819 (71.7)
35+	179 (15.7)
Missing	1 (0.1)
Ethnicity	
African descent	520 (45.5)
Asian descent	334 (29.2)
Other	285 (24.9)
Missing	4 (0.3)
Income (SRD)	
<3000	763 (66.8)
≥3000	333 (29.1)
Missing	47 (4.1)
Educational Level	
None, primary, lower secondary/vocational	658 (57.6)
Upper secondary/vocational or tertiary	485 (42.4)
Missing	47 (4.1)
Marital Status	
Not married/not living together	141 (12.3)
Married/cohabitating	1000 (87.5)
Missing	2 (0.2)
Parity (previous live births)	
0 (primiparity)	384 (33.6)
1	312 (27.3)
≥2	445 (38.9)
Missing	2 (0.2)
Region	
Urban	656 (57.4)
Rural	276 (24.1)
Interior	211 (18.5)
Missing	0 (0)

**Table 2:** Perceived stress, depression, and demographic characteristics by chemical stressor (mercury) (*n* = 827).

Characteristics	Hg Level ≥ 1.1 μg/g *n* (%)	Hg Level < 1.1 μg/g *n* (%)	OR [95% CI]	*p*-Value
Total *n* (%)	310 (37.5)	517 (62.5)		
Perceived stress				0.097
Low perceived stress	231 (39.1)	360 (60.9)	1	
High perceived stress	72 (32.7)	148 (67.3)	0.76 [0.55–1.05]	
Depression				0.355
No depression	235 (36.8)	404 (63.2)	1	
Probable depression	73 (40.6)	107 (59.4)	1.17 [0.84–1.65]	
Age (Years)				0.002
16–19	52 (49.5)	53 (50.5)	1.93 [1.27–2.93]	
20–34	201 (33.7)	395 (66.3)	1	
35+	56 (44.8)	69 (55.2)	1.60 [1.08–2.36]	
Ethnicity				0.001
African descent	122 (36.3)	214 (63.7)	1.43 [1.01–2.02]	
Asian descent	75 (28.5)	188 (71.5)	1	
Other	113 (50.0)	113 (50.0)	2.51 [1.73–3.64]	
Income (SRD)				0.001
<3000	246 (43.7)	317 (56.3)	2.67 [1.88–3.78]	
≥3000	53 (22.6)	182 (77.4)	1	
Educational level				0.001
None, primary or lower secondary	240 (50.2)	238 (49.8)	4.02 [2.93–5.52]	
Upper secondary or tertiary	70 (20.1)	279 (79.9)	1	
Marital status				0.002
Married/cohabitating	291 (39.4)	448 (60.6)	2.29 [1.35–3.89]	
Not married/not living together	19 (22.1)	67 (77.9)	1	
Parity (previous live births)				0.003
0 (primiparity)	100 (34.5)	190 (65.5)	1	
1	71 (31.3)	156 (68.7)	0.87 [0.60–1.25]	
≥2	138 (44.7)	171 (55.3)	1.53 [1.10–2.13]	
Region				0.001
Urban	96 (22.3)	334 (77.7)	1.27 [0.88–1.84]	
Rural	63 (26.8)	172 (73.2)	1	
Interior	151 (93.2)	11 (6.8)	47.76 [24.86–91.74]	

Hg = mercury, mean = 1.857 μg/g, median = 0.826 μg/g, range = 0–31.91 μg/g.

**Table 3:** Association between mercury, perceived stress, and depression, and adverse birth outcomes.

**Characteristic**	**LBW**	**LBW**	**OR (95%CI)**	***p*-Value**
	**Yes**	**No**		
	***n* (%)**	***n* (%)**		
Total	127 (13.2)	835 (86.8)		
Mercury				0.902
<1.1 μg/g	58 (13.3)	377 (86.7)	1	
≥1.1 μg/g	35 (13.0)	234 (87.0)	0.97 [0.62–1.53]	
Perceived stress				0.415
Low perceived stress	89 (12.9)	603 (87.1)	1	
High perceived stress	37 (14.9)	211 (85.1)	1.19 [0.79–1.80]	
Depression				0.733
No depression	97 (13.1)	641 (86.9)	1	
Probable depression	28 (14.1)	171 (85.9)	1.08 [0.69–1.70]	
	**PTB**	**PTB**		
	**Yes**	**No**		
	***n* (%)**	***n* (%)**		
Total	146 (15.2)	817 (84.8)		
Mercury				0.808
<1.1 μg/g	63 (14.5)	371 (85.5)	1	
≥1.1 μg/g	41 (15.2)	229 (84.8)	1.05 [0.69–1.62]	
Perceived stress				0.230
Low perceived stress	99 (14.3)	591 (85.7)	1	
High perceived stress	44 (17.5)	207 (82.5)	1.27 [0.86–1.87]	
Depression				0.953
No depression	114 (15.5)	623 (84.5)	1	
Probable depression	30 (14.9)	172 (85.1)	0.95 [.62–1.47]	
	**Low Apgar**	**Low Apgar**		
	**Yes**	**No**		
	***n* (%)**	***n* (%)**		
Total	31 (3.3)	918 (96.7)		
Mercury				0.242
<1.1 μg/g	17 (3.9)	416 (96.1)	0.57 [0.22–1.46]	
≥1.1 μg/g	6 (2.3)	258 (97.7)		
Perceived stress				0.053
Low perceived stress	18 (2.6)	665 (97.4)	1	
High perceived stress	13 (5.3)	234 (94.7)	2.05 [0.99–4.25]	
Depression				0.550
No depression	25 (3.4)	706 (96.6)	1	
Probable depression	5 (2.6)	190 (97.4)	0.74 [0.28–1.97]	

LBW = low birthweight, PTB = preterm birth.

**Table 4:** Multivariable model for low birthweight.

Variables	AOR (95% CI)	*p*-Value
Mercury		0.079
Low (1st quartile)	1	
High (4th quartile)	0.51 [0.24–1.08]	
Perceived stress		0.389
High perceived stress	1.41 [0.64–3.10]	
Low perceived stress	1	
Depression		0.777
No depression	1	
Probable depression	1.13 [0.47–2.71]	
Age (Years)		0.008
16–19	0.44 [0.14–1.37]	
20–34	1	
35+	3.15 [1.37–7.24]	
Educational level		0.020
None, primary, lower secondary/vocational	2.62 [1.11–6.18]	
Upper secondary/vocational or tertiary	1	
Parity		0.019
0 (nulliparous)	3.16 [1.29–7.73]	
1	1.06 [0.39–2.87]	
≥2	1	
Mercury × perceived stress *	0.65 [0.12–3.51]	0.612
Mercury × depression *	0.67 [0.12–3.90]	0.655

* Interaction between mercury and stress/depression.

**Table 5:** Multivariable model for preterm birth.

Variables	AOR (95% CI)	*p*-Value
Mercury		0.039
Low (1st quartile)	1	
High (4th quartile)	2.47 [1.05–5.83]	
Perceived stress		0.443
High perceived stress	1.36 [0.62–2.96]	
Low perceived stress	1	
Depression		0.164
No depression	1	
Probable depression	0.49 [0.18–1.33]	
Age (Years)		0.733
16–19	1.36 [0.58–3.23]	
20–34	1	
35+	1.19 [0.49–2.90]	
Ethnicity		0.268
African descent	1.32 [0.52–3.37]	
Asian descent	1	
Other	2.11 [0.79–5.63]	
Parity		0.525
0 (nulliparous)	1.53 [0.66–3.54]	
1	1.56 [0.67–3.60]	
≥2	1	
Region		0.197
Urban	0.75 [0.31–1.80]	
Rural	1	
Interior	0.38 [0.13–1.09]	
Mercury × perceived stress *	0.33 [0.06–1.76]	0.195
Mercury × depression *	5.90 [0.52–66.70]	0.152

* Interaction between mercury and stress/depression.

**Table 6:** Multivariable model for low Apgar score.

Variables	AOR (95% CI)	*p*-Value
Mercury		0.339
Low (1st quartile)	1	
High (4th quartile)	2.49 [0.38–16.20]	
Perceived stress		0.004
High perceived stress	9.73 [2.03–46.70]	
Low perceived stress	1	
Depression		0.434
No depression	1	
Probable depression	0.47 [0.07–3.09]	
Ethnicity		0.183
African descent	6.05 [0.65–56.43]	
Asian descent	1	
Other	1.53 [0.09–27.03]	
Parity		0.720
0 (nulliparous)	1.53 [0.66–3.54]	
1	1.56 [0.67–3.60]	
≥2	1	
Region		0.728
Urban	2.48 [0.27–23.26]	
Rural	1	
Interior	0.00 [0.00–0.00]	
Mercury × perceived stress *	0.61 [0.01–45.64]	0.823
Mercury × depression *	2.56 [0.03–244.22]	0.686

* Interaction between mercury and stress/depression.
